# Safety and effectiveness of ataluren in patients with Duchenne muscular dystrophy: single-center experience from Saudi Arabia

**DOI:** 10.1177/03000605241305252

**Published:** 2024-12-24

**Authors:** Mushtaha Ahmad, Alaa ElRasoul, Raneem Sedayou, Mohammed Tamboosi, Hanan Mahroos, Shaimaa Alrashed, Mariam Tunkar, Faisal Alzahrani, Mohammed Alharbi, Mona Aljehani, Mousa Alahmari, Khalid Alqarni, Maha Gashlan, Berna Seker Yilmaz, Nahla M. Alshaikh

**Affiliations:** 1 Department of Pediatrics, 47798 Ministry of National Guard Health Affairs, Jeddah, Saudi Arabi; 2 Rehabilitation Center, 47798 Ministry of National Guard Health Affairs, Jeddah, Saudi Arabia; 3 Pharmaceutical Department, 47798 Ministry of National Guard Health Affairs, Jeddah, Saudi Arabia; 4 College of Medicine, 48149 King Saud bin Abdulaziz University for Health Sciences, Jeddah, Saudi Arabia; 5 King Abdullah International Medical Research Center, Jeddah, Saudi Arabia; 6 UCL, Great Ormond Street Institute of Child Health, London, UK

**Keywords:** Ataluren, dystrophin, nonsense mutation Duchenne muscular dystrophy, loss of ambulation, case series, 6-minute walking distance, ambulation

## Abstract

**Objective:**

Duchenne muscular dystrophy (DMD) is a rare X-linked neurodegenerative disorder caused by mutations in the *DMD* gene. This study examined the efficacy and safety of ataluren, the first oral treatment for DMD with nonsense mutations (nmDMD), in patients in the Middle East.

**Methods:**

This retrospective longitudinal study assessed the outcomes of seven boys with nmDMD who received treatment with ataluren and follow-up at a single center since 2016.

**Results:**

The median patient age at treatment initiation was 8.04 years (range: 3.3–9.92), and the median duration of exposure was 3.95 years (interquartile range = 4.42 years). Five patients were still ambulatory at the last follow-up. Ataluren was more effective in individuals with baseline 6-min walking distance (6MWD) ≥300 m, as these patients had smaller declines in 6MWD and North Star Ambulatory Assessment scores. Pulmonary function was well preserved in all patients, with no patients having forced vital capacity <60% at their last follow-up. Six patients maintained normal cardiac function, whereas one patient developed heart failure before starting ataluren treatment.

**Conclusions:**

Our results demonstrated both the efficacy and safety of ataluren. Early initiation of ataluren treatment delayed the loss of ambulation and cardiorespiratory milestones.

## Introduction

Duchenne muscular dystrophy (DMD; OMIM 310200) is a rare, degenerative X-linked neuromuscular disorder that occurs in approximately 1 in 3500 to 5000 live male births with a pooled global prevalence of 4.78 per 100,000 (95% confidence interval [CI] = 1.9–11.8).^1–3^ DMD is associated with progressive muscle-wasting caused by mutations in the *DMD* gene that prevent the production of the functional muscle isoform of dystrophin (Dp427m).^4^ Thousands of different mutations have been reported in the *DMD* gene. Almost 60% to 70% of mutations are deletions, 5% to 15% are duplications, and 20% are point mutations, small deletions, or insertions.^5^

Patients with DMD often present with delays in motor milestones and frequent falls in early childhood.^2^ Because of the continuous decline in muscle function, patients experience a loss of ambulation, respiratory complications, and cardiomyopathy. Most patients become wheelchair users around 10 years of age, and they require respiratory support around 20 years of age.^6^ Premature death occurs by the second to fourth decade of life based on clinical management and the disease phenotype.^7^

Currently, there is no cure for DMD. The current standard of care (SoC) includes long-term corticosteroid therapy, which delays the loss of ambulation and slows the decline in upper-limb and respiratory functions, but this treatment can cause significant side effects.^4,8,9^ Meanwhile, ataluren is the first targeted therapy approved for patients with nonsense mutation Duchenne muscular dystrophy (nmDMD).The drug, which was conditionally approved by the European Medicines Agency in 2014.^10^, can slow disease progression by restoring dystrophin production in patients with nmDMD *via* read-through of premature stop codons during protein translation.^11^

The efficacy of ataluren was evaluated in two previous randomized, placebo-controlled studies: PTC124-GD-007-DMD (NCT00592553) and PTC124-GD-020-DMD (NCT01826487). Although the results for the primary endpoints were not statistically significant in these two studies, ataluren was well tolerated, and it slowed the decline in motor function at a dose of 40 mg/kg/day.^10,12^ A Phase III long-term study found that in addition to delaying the age of loss of ambulation, ataluren also slows respiratory deterioration in both ambulatory and non-ambulatory patients with nmDMD (NCT01557400).^13^ Data from an on ongoing multicenter registry (Strategic Targeting of Registries and International Database of Excellence [STRIDE], NCT02369731) illustrated the efficacy of ataluren combined with SoC in delaying the decline of motor and respiratory function compared with SoC alone.^14,15^

A recent Phase III randomized (1:1), double-blind, placebo-controlled trial (NCT03179631) enrolled 359 patients aged 7 years or older with nmDMD. The trial included a 72-week double-blind phase, followed by a 72-week open-label extension in which patients who had received placebo were switched to ataluren. The primary endpoint was the slope of the change from baseline in the 6-minute walking distance (6MWD) at 72 weeks. In patients with deterioration of ambulation, the mean change in 6MWD from baseline was −81.83 m in the ataluren group, versus −90.09 m in the placebo group, giving a non-significant difference of 8.26 m (95% CI = −26.05 to −9.53, *P* = 0.36). However, differences in the mean 6MWD change from baseline and the rate of variation favored ataluren in the intention-to-treat (ITT) population (14.4 m; 0.20 m/week; *P* = 0.0248) and the 300- to 400-m baseline 6MWD subgroup (24.2 m; 0.34 m/week; *P* = 0.0310), which experienced decreases of 21% and 30% in the decline rate of 6MWD, respectively. Meanwhile, the risk of 10% persistent worsening in 6MWD was 31% (*P = *0.0078) and 47% (*P* = 0.0011) lower in the ITT population and baseline 6MWD ≥300 m to <400 m subgroup, respectively, compared with placebo. The number of ITT patients with loss of ambulation was almost 2-fold higher in the placebo group than in the ataluren group.^16^

Ataluren has been a registered treatment in the Kingdom of Saudi Arabia since April 2021, and the drug is currently prescribed in this country. The present study investigated the long-term outcomes of our patients with DMD treated with ataluren, focusing on motor, respiratory, and cardiac function.

## Materials and methods

### Study population

This retrospective, longitudinal case series investigated boys with nmDMD who have been treated with ataluren and followed-up at the Department of Pediatrics, Ministry of National Guard Health Affairs (Jeddah, Saudi Arabia) since 2016. All patients had a genetically verified nonsense mutation leading to a premature stop codon. Both ambulatory and non-ambulatory patients were involved. The reporting of this study conforms to the CARE guidelines.^17^

### Data collection

Data were retrospectively collected from the patients’ medical records using a case report form, which is available upon request. The data included age at symptom onset, age at corticosteroid and ataluren initiation, ambulation status, family history, results of diagnostic testing, 6MWD, functional motor abilities, upper limb function, and respiratory and cardiac parameters.

### Data elements

A physical examination including a complete neurological examination and history taking from the time of the previous visit was performed every 24 weeks by a pediatric neurologist.

The 6-minute walking test (6MWT) was performed in ambulatory patients. The test was performed indoors on a flat, non-slippery surface with marked cones indicating the 30-m walking path. Patients were instructed to complete laps within 6 minutes at their normal pace, with the option to rest or use walking aids. The total distance covered was calculated by counting complete 30-m walks and any remaining partial laps.

Functional motor abilities were assessed using the North Star Ambulatory Assessment (NSAA), a 17-item, validated functional rating scale specifically developed for measuring motor function in ambulatory patients with DMD. Each item on the NSAA was scored between 0 and 2, giving a maximum total score of 34. For each item, the score was awarded as follows: 0, unable to perform independently; 1, able to perform with assistance; and 2, normal, able to perform without assistance.^18^

Upper limb function was assessed using the performance of upper limb (PUL) assessment at high (shoulder), middle (elbow), and distal (wrist and hand) levels. The assessment was conducted using the PUL 2.0 scoresheet.^19^

Pulmonary function assessment was performed by spirometry using a HypAir PFS (MGC Diagnostics, Saint Paul, MN, USA). The evaluated parameters were forced vital capacity (FVC) and forced expiratory volume in 1 s (FEV1).

Cardiac function was analyzed by echocardiography using a Philips Diagnostic Ultrasound System Model (Philips, Amsterdam, Netherlands). A transducer probe was used to capture real-time images of the heart by scanning across the chest and evaluate parameters of cardiac function, including ejection fraction (EF) and fractional shortening (FS).

### Ethical statement

This study was approved by the Institutional Review Board (IRB) of King Abdullah International Medical Research Center (approval number, IRB/0731/24; study number, NRJ23J/352/12). Because patient data were retrospectively analyzed, the requirement for written informed consent was waived according to the local ethics guidelines. All patient details have been de-identified, ensuring anonymity.

### Statistical analysis

The statistical evaluations were descriptive. Because of the small number of patients, frequencies were presented as medians and ranges. Histograms and scatter plots were used to visualize the data distribution and illustrate differences among patients.

## Results

In total, seven boys with a confirmed nonsense mutation in the *DMD* gene were included in this study. Patients’ disease-specific characteristics and genetic findings are summarized in Table 1. All patients were of Saudi Arabian descent. Six patients were born to consanguineous parents, and two patients had a family history.

**Table 1.** Patients’ demographic, clinical, and genetic findings.

Patient No.	Consanguinity	Family history	Current age (years)	First symptoms	Age of first symptoms (years)	Age of diagnosis (years)	Mutation
1	Yes	No	11.83	Frequent falls and difficulties regarding jumping and running	2	7	c.1346T>A(p.Leu449*)
2	Yes	Sibling	7.18	No symptoms	N/A	2	c.6502G>T (p.Glu2168*)
3	Yes	No	6.42	Limited motor abilities	5	5	c.5344G>T(p.Glu1782*)
4	Yes	No	11.99	Frequent falls and difficulties with stairs	2	7.25	c.721C>T(p.Gln241*)
5	Yes	No	14.24	Limited motor abilities with easy fatigability and difficulties in standing from a sitting position	8	8	c.4729C>T(p.Arg1577*)
6	No	Maternal first-degree cousin had suspicion of DMD	11.21	Frequent falls and difficulty in running	5	5	c.721C>T(p.Gln241*)
7	Yes	No	14.39	Frequent falls, waddling gait, with difficulties with walking and running	2	8.16	c.4897G>T(p.Glu1633*)

DMD, Duchenne muscular dystrophy

The most common initial symptoms were frequent falls and difficulties with walking, running, and jumping. The median ages at first symptoms and genetic confirmation of the nmDMD diagnosis were 3.5 (2–8) and 7 years (2–8.16), respectively. Most patients were diagnosed after having symptoms, although one patient was diagnosed in a pre-symptomatic state because of an index case in the family (patient 2).

Patients’ treatment overview and ambulation status are highlighted in Table 2. The mean patient age at ataluren treatment initiation was 7.51 ± 2.22 years (median, 8.04 years; range, 3.3–9.92). All seven patients are currently receiving treatment with ataluren in combination with corticosteroids, specifically prednisolone. The median duration of exposure to ataluren was 3.95 years (interquartile range: 4.42 years). Five patients were already receiving corticosteroids at the time of ataluren treatment initiation, corticosteroids and ataluren were initiated simultaneously in one patient (patient 4), and corticosteroids were initiated 2.48 years after ataluren treatment in one patient (patient 2).

**Table 2.** Treatment and ambulation status overview.

Patient No.	Age at ataluren initiation (years)	Age at corticosteroid initiation (years)	Ambulation status at the time of ataluren initiation	Duration of ataluren exposure (years)	Duration of corticosteroid exposure (years)	Age at loss of ambulation (years)	Ambulation status at last visit
1	7.41	6.87	Ambulatory	4.42	4.96	N/A	Ambulatory
2	3.3	5.78	Ambulatory	3.88	1.4	N/A	Ambulatory
3	6.28	5.62	Ambulatory	0.14	0.8	N/A	Ambulatory
4	8.04	8.04	Ambulatory	3.95	3.95	N/A	Ambulatory
5	9.92	7.65	Ambulatory	4.32	6.59	13	Non-ambulatory
6	8.2	6.72	Ambulatory	3.01	4.49	N/A	Ambulatory
7	9.47	8.54	Ambulatory	4.92	5.85	11.8	NA

N/A: not available

### Motor function outcomes

Five patients were still ambulatory at the last follow-up. The other patients lost ambulation at the ages of 13 and 11.8 years, respectively (patients 5 and 7, respectively; Table 2). These two patients started ataluren treatment at the latest ages (9.92 and 9.47 years, respectively), and they became non-ambulatory after 3.08 and 2.33 years of treatment, respectively. The five patients who started ataluren treatment at younger ages (<9 years) remained ambulatory at a median age of 11.21 years (6.42–11.99).

**Figure 1.**
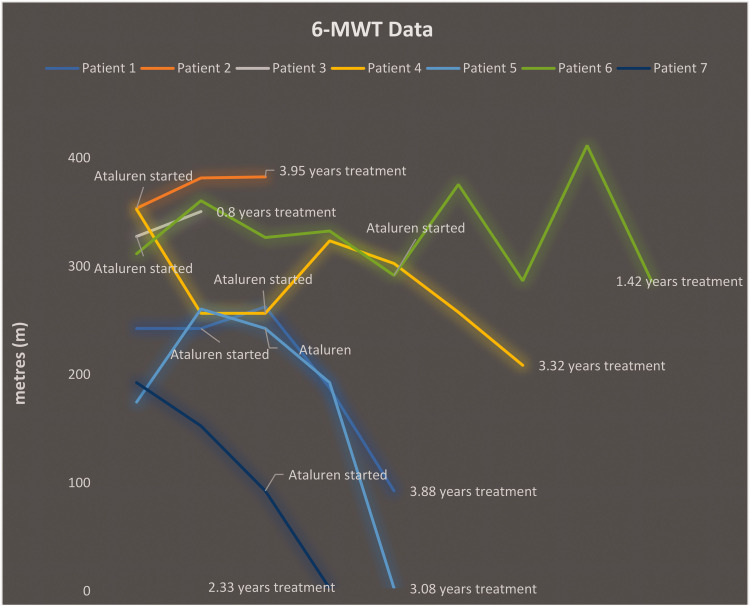
Six-minute walking test results in patients with Duchenne muscular dystrophy.

6MWT data were available for all patients after ataluren initiation, and the findings are summarized in Figure 1. Patient 2, who started ataluren treatment prospectively without having any symptoms because of an index case in the family, displayed an 8.2% increase in 6MWD over a 1.48-year period of ataluren treatment, with the improvement then remaining stable. Ataluren treatment was more effective in individuals with a baseline 6MWD of at least 300 m, as these patients had a smaller decline in 6MWD, which remained above 200 m at the last follow-up.

NSAA scores are reported in Table 3. Scores after starting ataluren treatment were only available for five patients. Of these five patients, the NSAA score initially increased or stabilized in four patients (patients 1, 2, 4, and 6). Meanwhile, patient 7 exhibited a decline in NSAA scores under ataluren treatment.

**Table 3.** NSAA scores in patients with DMD.

Patient No.	Age at ataluren initiation (years)	NSAAAge (years)/score						
1	7.41	6.87/32	8.10/19	9.71/22	10.77/22	11.94/18		
2	3.3	5.53/28	6.11/34	7.01/34				
3	6.28	5.94/33						
4	8.04	7.37/31	8.31/30	9.41/30	10.4/29	10.82/24	11.85/20	
5	9.92	9.32/20	9.54/19					
6	8.2	6.67/33	6.86/33	7.47/31	8.11/28	8.67/30	9.32/29	9.56/25
7	9.47	8.54/27	9.04/22	11.5/10				

NSAA, North Star Ambulatory Assessment.

PUL scores were only available for two patients (patients 5 and 7), with the test performed during ataluren therapy in both patients. The PUL score was 30 in both patients, and upper limb function was preserved in both ambulatory and non-ambulatory patients.

### Pulmonary function

**Figure 2.**
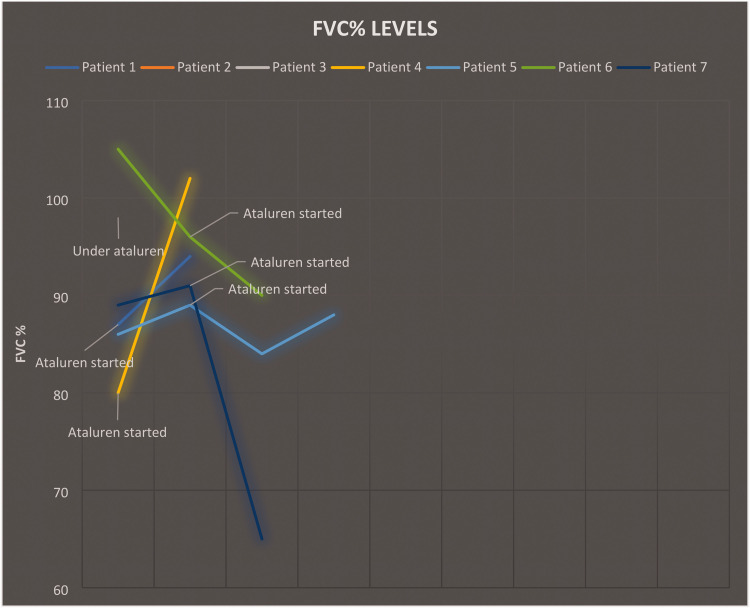
Changes in FVC during ataluren treatment. FVC, forced vital capacity.

Pulmonary follow-up data were available for six patients, and ataluren treatment appeared to slow the decline of respiratory function compared with that expected naturally in similar patient populations without treatment. No patients had FVC < 60% at their last follow-up. Additionally, FVC remained higher than 80% in five of six patients, suggesting that ataluren can help maintain respiratory function over time (Figure 2).

The results of sleep studies were normal during follow-up in six patients, whereas one patient (patient 1) developed obstructive sleep apnea at 10.07 years old and required non-invasive ventilation.

### Cardiac functions

Six patients had normal cardiac function at their last follow-up. One patient (patient 5) developed heart failure at 9.25 years old (EF = 31%), and he experienced an acute myocardial injury at 10.85 years old. Three patients (patients 2, 5, and 7) are currently on angiotensin-converting enzyme inhibitor treatment.

### Safety

Ataluren treatment was safe and well tolerated throughout the follow-up period without any reported side effects, whereas weight gain (two patients) and osteoporosis (two patients) were the two most common side effects associated with corticosteroids.

Compliance to treatment was extremely high, and no patients have discontinued treatment.

## Discussion

DMD is a severe neuromuscular disorder resulting from deficiency of the functional dystrophin protein.^4^ Ataluren promotes the production of full-length dystrophin *via* read-through of an in-frame premature stop codon, and it is indicated for the treatment of patients with nmDMD.^20,21^ To the best of our knowledge, this is the first report of long-term ataluren treatment in patients with nmDMD from the Kingdom of Saudi Arabia. Our results highlighted the positive effects of ataluren on the loss of ambulation and motor function as well as pulmonary and cardiac involvement.

Although the median age at the first presentation was 3.5 years, the median age at diagnosis was 7 years, highlighting a 3.5-year delay between the first symptom and diagnosis in our patients. A recent observational study in the Kingdom of Saudi Arabia also reported a median age of diagnosis of 7 years old.^22^ Muscular Dystrophy Surveillance, Tracking, and Research Network data revealed a 2.5-year delay between symptom onset and definitive diagnosis over the previous two decades.^23^ However, differences have been observed between countries, as the time between first symptoms and diagnosis was reported as 7 months in Austria and 4.7 months in Germany.^24^

Early treatment initiation is important to prevent progressive muscle loss and rapid disease progression.^25,26^ In our cohort, the median age at ataluren initiation was 8.04 years, earlier than that in either the STRIDE registry or Swedish cohort, which reported ages of 9.8 and 8.6 years, respectively.^14,27,28^

Two of our patients lost ambulation at the ages of 11.8 and 13 years, respectively, which were later than that (11 years) reported in the Cooperative International Neuromuscular Research Group (CINRG) Duchenne Natural History Study (DNHS). The median ages at loss of ambulation for the matched populations from the STRIDE Registry and the CINRG DNHS were 14.5 (95% CI = 13.9–NA) and 11.0 years (95% CI = 10.5–12.0), respectively. This illustrates a statistically significant benefit for ataluren (*P* < 0.0001; hazard ratio =0.283).^14^ In our cohort, the two patients who became non-ambulatory started ataluren treatment at an older age than the other patients. Patients who started ataluren treatment before turning 9 have remained ambulatory. Early initiation of ataluren treatment was also associated with better outcomes in a Swedish cohort. Patients who started ataluren at earlier years were found to be ambulatory at a median age of 14.4 years.^28^ Improvement in motor function was best in one of the three Italian patients who started ataluren treatment at the youngest age.^29^

In this study, ataluren was more effective in patients with baseline 6MWD in the range of ≥300 to <400 m, and these patients had a smaller decline in 6MWD, which remained higher than 200 m at the last follow-up. Previous reports also revealed the significant effect of ataluren in the same subgroup of patients compared with patients higher or lower baseline 6MWD.^10,30^ A recent meta-analyses demonstrated that ataluren has the most prominent effect in patients with a baseline 6MWD of ≥300 to <400 m versus placebo over a 48-week period.^31^

Previous trials described the beneficial effect of ataluren on upper limb function. The drug preserved hand-to-mouth function by 3.4 years compared with the findings in the natural history group. The effect on PUL score changes was also more significant in the 300- to 400-m 6MWD subgroup.^32^ In our patients, a review of medical records also highlighted that upper limb function was well preserved in all patients. However, the PUL score was only available for two patients. Although both patients were non-ambulatory on the test day, they both had a high PUL score of 30.

There was an initial increase/stabilization in NSAA scores among our patients with baseline 6MWD > 200 m. NSAA scores decreased during ataluren treatment only in patient 7. This patient had baseline 6MWD < 200 m. The overall findings were in line with previous trials, in which patients who received ataluren achieved a 1.5-point improvement in the NSAA score compared with patients who received placebo (mean NSAA score: −7.0 vs. −8.5; *P* = 0.270).^33^ The benefit of ataluren was more pronounced among patients with baseline 6MWD of 300 to 400 m, for whom ataluren was associated with a 4.5-point improvement in the NSAA score versus placebo (mean observed NSAA score: −5.7 vs. −10.2; *P* = 0.030).^34^

We propose that ataluren delays pulmonary involvement in patients with nmDMD. Although the follow-up duration varied among our patients, none had FVC < 60% at the last follow-up. This outcome is consistent with previous studies, which reported that ataluren plus SoC provided a 3-year delay in the predicted decline of FVC to lower than 60% compared with SoC alone.^13^ This effect was more remarkable in ambulatory patients in a Swedish cohort, who experienced an median annual increase in predicted FVC of 4.19 per year (2.8%–8.2%).^28^ The latest report from the STRIDE Registry indicated that ataluren plus SoC delayed the age of predicted FVC < 60% by 1.8 years (*P* = 0.0021) and delayed predicted FVC < 50% by 2.3 years (*P* = 0.0207). The annual changes in predicted FVC decline for non-ambulatory patients were −3.07 and −3.95 in the STRIDE Registry and CINRG DNHS, respectively (*P* = 0.0854). Although the difference was not statistically significant, it was obvious that ataluren has also a favorable effect on pulmonary function in non-ambulatory patients.^15^ Similarly, ataluren improved FVC in three of four non-ambulatory patients with DMD in a study from Germany.^35^

Cardiac function was well preserved in our DMD cohort, as only one patient developed heart failure. This patient started ataluren at a later age (9.92 years) than the remaining patients, and heart failure occurred before he initiated ataluren treatment. Limited data have been published on cardiac function during ataluren therapy. Ataluren plus SoC delayed the deterioration of cardiac function compared with SoC alone, although the effect was not statistically significant.^15^ A recent report of ataluren treatmetn in non-ambulatory patients revealed that ataluren improved FS in three of four patients.^35^

In our cohort, although patients experienced several side effects associated with corticosteroid treatment, ataluren was well-tolerated without any side effects. Previous reports also indicated ataluren was safe and well accepted with high compliance.^10,12,14,35,36^

The limitations of our study included its small cohort with a broad age range, and patients were heterogenous in terms of disease stages, treatment initiation, and treatment duration. This cohort did not include a control group, but we compared our results with those of the CINRG DNHS study. Although comparing our findings with registry data helps to contextualize our results, incorporating a matching or statistical approach to account for differences in patient characteristics would strengthen the validity of this comparison. Unfortunately, we could not implement such an approach because of the small number of patients in our cohort, as well as the variability in the follow-up periods and available data elements.

There is strong evidence that ataluren slows disease progression in patients with nmDMD. Therefore, ataluren treatment significantly improves patients’ quality of life by maintaining their motor function and preventing cardiorespiratory complications. Maintaining independence and the ability to perform simple daily activities is meaningful and important for patients and their families.^30^

In conclusion, although our results suggest a potential benefit of ataluren in delaying the loss of ambulation, preserving upper limb function, and slowing the deterioration of respiratory and cardiac function, the study’s limitations must be considered. Given the small sample size and the lack of a control group, these findings should be interpreted with caution. We propose that ataluren offers promise for patients with DMD, particularly if treatment is started early and continued after the loss of ambulation to prevent further complications. However, additional research, including statistical analyses and comparisons with historical controls, is necessary to validate these findings and more definitively establish ataluren’s role in DMD management. As there remains an unmet need for effective DMD treatments, ataluren’s potential effects on patients’ lives warrant further investigation.

## Data Availability

The data that support the findings of this study are available from the corresponding author upon reasonable request.
